# Monsel’s Salt

**Published:** 1859-05

**Authors:** 


MONSEL'S SALT.—We have lately received several letters relative to the preparation of this new remedy, and we give the formula as it is found in Monsel's article in the Journal de Pharmacie et de Chimie, September, 1857. We prefer giving the French weights, as we could only approximate them by reducing them to the English standard. Every accomplished druggist, who alone is able to make these articles, should have his box of French weights whenever he enters upon the preparation of an article so important as this.

Distilled water, one hundred grammes;

Sulphuric acid, at 60° [Baumé,] ten grammes.

Carry to ebullition in a porcelain capsule of a demi-litre capacity, and add

Protosulphate of iron, fifty grammes.

After complete solution, add gradually to the boiling liquid,

Nitric acid, at 35° [Baumé,] sixteen grammes.

And when the disengagement of vapors has ceased, add gradually,

Powdered protosulphate of iron, fifty grammes.

Complete the bulk of one hundred grammes by adding distilled water. Let cool and filter.

The salt crystallizes in scales of a brownish-red color, has an astringent taste, and no caustic properties. As a hemostatic and astringent, the soluble persulphate of iron is beginning to have a wide reputation, and when applied to a bleeding surface, it produces a large and firm clot, which increases in hardness for several hours. We have found it useful in cases of nervous and other affections. The preparation can be obtained of Mr. Hubbell, an accomplished pharmaceutist of this city.

